# P-1815. Estimation of the cases with suspected community-acquired pneumonia where antibiotic intervention could occur after 48 hours: Application of an electronic algorithm for real-time screening

**DOI:** 10.1093/ofid/ofae631.1978

**Published:** 2025-01-29

**Authors:** Bongyoung Kim, Eili Klein, Valeria Fabre, Sara E Cosgrove

**Affiliations:** Department of Internal Medicine, Hanyang University College of Medicine, Seongdong-gu, Seoul-t'ukpyolsi, Republic of Korea; Johns Hopkins School of Medicine, Baltimore, Maryland; Johns Hopkins University School of Medicine, Baltimore, MD; Johns Hopkins School of Medicine, Baltimore, Maryland

## Abstract

**Background:**

Timely identification of unnecessary antibiotics (abx) started for suspected community-acquired pneumonia (CAP) is vital for antibiotic stewardship programs (ASPs). We estimated potential opportunities for ASP intervention among patients flagged as potentially having CAP by an electronic algorithm using electronic health record data that allows for real-time case detection.

**Figure:**
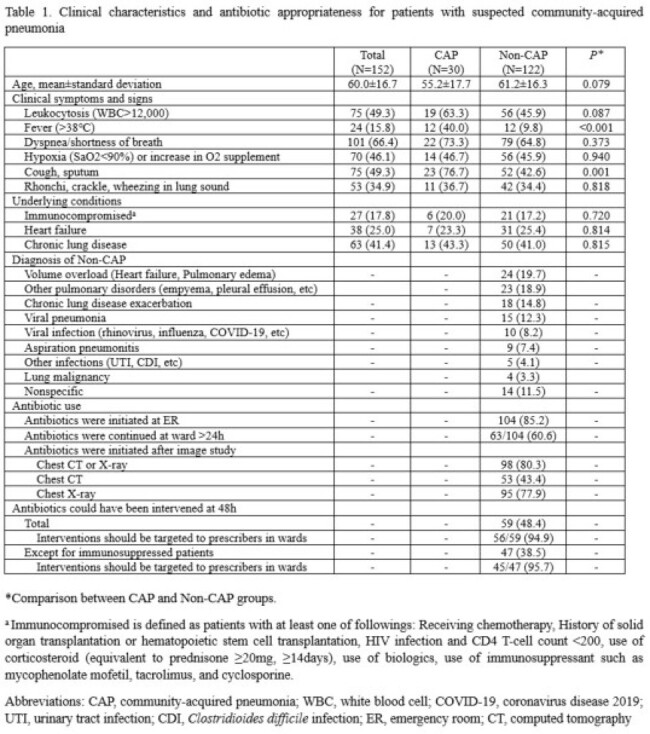

**Methods:**

We screened all adult patients admitted to Johns Hopkins Hospital (1,150 beds) between 12/2023-2/2024 using the algorithm which is based on the following variables: i) abx targeting CAP ≥48h, ii) respiration rate ≥24/min, iii) white blood cell (WBC) >12,000/mm^3^, and iv) natural language processing (NLP)-derived radiology results compatible with pneumonia. Patients admitted to oncology or with sickle cell disease were excluded. Manual chart review was performed to adjudicate true CAP, defined as the presence of signs/symptoms of pneumonia with corresponding radiographic findings, while excluding alternative diagnoses, based on the clinical course observed within 48 h. We characterized factors associated with patients without CAP, particularly radiological interpretation, and the proportion eligible for ASP intervention at 48 h of admission.

**Figure:**
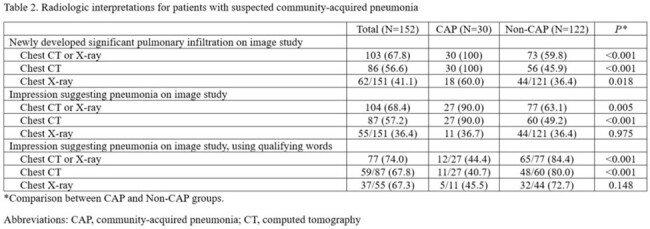

**Results:**

122 of the 152 cases (80.3%) identified by the electronic algorithm were adjudicated as non-CAP based on clinical criteria. Compared to CAP cases, non-CAP cases were less likely to have fever (40.0% vs. 9.8%, *P* < 0.001) and cough/sputum (76.7% vs. 42.6%, *P* = 0.001). Common diagnoses for non-CAP cases were volume overload (19.7%), other pulmonary disorders (18.9%), chronic lung disease exacerbation (14.8%), and viral pneumonia (12.3%).

Most unnecessary abx were initiated in the emergency room (85.2%, 104/122); 60.6% (63/104) were continued for >24 hours after admission, and 48.4% (59/122) could have been intervened upon at 48 hours of admission.

63.1% of non-CAP cases had radiographic interpretations suggesting CAP, with poor correlation between radiological interpretation and clinical diagnosis of true CAP (kappa 0.221).

**Figure:**
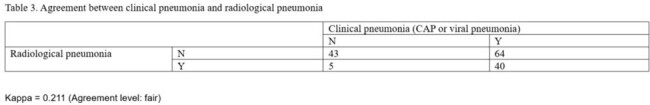

**Conclusion:**

ASP interventions to stop abx within 48 h of admission were indicated in ∼50% of non-CAP cases identified by the electronic algorithm.

**Disclosures:**

**All Authors**: No reported disclosures

